# Correlations between the Major Amino Acids and Biochemical Blood Parameters of Pigs at Controlled Fattening Duration

**DOI:** 10.3390/molecules27072278

**Published:** 2022-03-31

**Authors:** Sergei Yu. Zaitsev, Nikita S. Kolesnik, Nadezhda V. Bogolyubova

**Affiliations:** Federal Research Center for Animal Husbandry Named after Academy Member L.K. Ernst, Dubrovitsy 60, 142132 Podolsk, Moscow Region, Russia; kominisiko@mail.ru (N.S.K.); 652202@mail.ru (N.V.B.)

**Keywords:** amino acids, amperometric method, biochemical parameters and methods, blood, chromatography, pig serum

## Abstract

Analytical control of protein and amino acid (AA) contents of animal tissues is an important problem in the fundamental and applied aspects. The aims of the work were the following: to measure the pig blood AAs; and to establish the correlations between AAs and biochemical parameters in dependence on the pig fattening duration. All 80 animals were divided onto 4 animal groups: 65, 72, 82, and 90 fattening days. The correlations between AAs and the total protein or its fractions (TP&F), nitrogen metabolites, carbohydrates, lipids, some enzymes in the pig blood for each of these animal groups obtained for the first time. The authors established the following total amounts of correlation coefficients (with reasonable *p*-values) in each of the group separately: group 1, 1* (*p* < 0.05); group 2, 0; group 3, 28* (*p* < 0.05) and 9** (*p* < 0.01); group 4, 28* (*p* < 0.05) and 25** (*p* < 0.01). Thus, about 82–90 days (groups 3 and 4) can be the optimal for the pig fattening, based on the correlation analysis for the numerous data of major AA and biochemical parameters of pig blood. These results can be useful for animal health monitoring and husbandry.

## 1. Introduction

Amino acids are performing important functions in the body as the major components of protein metabolism [1,2,3,4,5]. First of all, it is necessary to measure the amount of essential amino acids (EAAs) such as tryptophan, phenylalanine, lysine, arginine, histidine, threonine, methionine, leucine, isoleucine and valine, which cannot be synthesized in the human body and should only be supplied with diet [6,7,8,9]. The calculated ratio of the EAAs to the so-called “synthesizable” (among the 20 general) amino acids (SAAs) is also important. The authors preferred such abbreviation as SAAs in comparison to so-called names as “non-essential” or “non-valuable” AAs, since all the 20 AA types must be available to ribosome for protein synthesis (i.e., all of these AAs are essential and valuable).

It has been found that a number of branched-chain amino acids (i.e., isoleucine, leucine, and valine) are “improving the quality of pig meat”, promoting muscle tissue growth, enhancing intestinal development, and “regulating the immune response” [10]. It is well known that free amino acids and many their derivatives can be an “extraordinary regulating factor” for connecting of the main metabolic processes [10,11,12]. For example, the metabolites of alanine, 4-hydroxyproline, tyrosine, methionine, etc. are associated with the Krebs cycle, which is a binder for many metabolic pathways [6,7,8]. Additionally, it was found [13,14] that a certain amount of copper (in the pig’s liver) significantly affects the concentration of some amino acids (leucine, phenylalanine, methionine, proline, tyrosine) in the animal blood serum, assuming a particular effect on the animal development [13,14]. Therefore, the development of scientifically proved “norms of the need for amino acids” is very important in the organization of rational nutrition for farm animals [15]. The authors of [12] considered that the most important amino acids for pigs are lysine, methionine, threonine, and tryptophan [12].

Among the 20 essential amino acids one can single out, for example, glutamine. The lack of this amino acid leads “to impaired immunogenesis” [12,16]. It is well known that amino acids are not accumulated in tissues, but are constantly present in the blood, while some are of exogenous origin; some are formed due to the breakdown of body proteins [17]. It is important to highlight that there are no clear standards for the content of free amino acids in the studied animal species [2,3,4,17,18,19]. In [12], the content of various amino acids in the blood serum of pigs “of early maturing meat breed” was measured [12]. For example, a relative amount of all amino acids in this case was 3.521 ± 0.252, which is slightly lower than the usually cited by about 5% [12].

In the papers of Morozova L.A. et al. [9] and Petukhova M.A. et al. [18] the comparable data on the content of free amino acids in pig meat were published. Petukhova M.A. et al. [18] investigated the amino acid composition of the meat of pigs of several breeds, including Duroc, while Morozova L.A. et al. [9] studied the content of AA in the muscle tissue of hybrid pigs (Landras·Yorkshire·Duroc). For example, the valine content in hybrid pigs was significantly lower than that of purebred Duroc (0.985 g/100 g and 1.460 g/100 g, respectively [9,18]), and the lysine content was 2.128 g/100 g for hybrid pigs [7,16] and 2.431 g/100 g—for purebred ones [9,18]. In turn, phenylalanine, histidine, threonine, methionine and tryptophan were found in almost equal amounts [18,19].

The interesting data on AA contents in pig blood were obtained by addition of three branched-chain amino acids (BAAs) as a part of different protein-restricted diets [20,21,22]. These observations suggested as a “dietary strategy to reduce N excretion from swine” [20], due the “N excretion from swine”, about 24–103 kg per 450 live weight per year. Moreover, the obtained AAs data were important for regulation of the central and peripheral factor’s influences on the growth rates of pigs fattening in the case of different protein-restricted diets [20,21,22]. The numerous data and recommendations concerning various diets for pig growth, fattening, etc. are summarized in the recent official documents [23,24], but this was not the main point of our research and was used to prove the correct protocol and importance of our study.

There are practically no works on a detailed study of the correlations between the amino acid composition and biochemical parameters of Duroc pig blood, while the study of correlations between the biochemical parameters itself of numerous blood samples of some pig breeds and various another animals is carried out all over the world [25,26,27,28,29,30]. For example, correlations have been found between serum biochemical parameters and amino acids “in piglets by early-weaning and proline and putrescine supplementations” [25]; correlation among serum biochemical indices and “slaughter traits, texture characteristics and water-holding capacity of sheep” [26]; correlation of different biochemical parameters “in blood sera of healthy and sick cows” [27]; correlations between states of amino acids and “hematology or plasma biochemistry in calves within 24 h after birth” [28]; and correlations between blood parameters “in fattening pigs from two genetic types fed diet with three different protein concentrations” [29]. In addition, an interesting comparative study of the “hematological, chemical and functional characteristics of porcine, chicken and duck blood” was performed [30]. The authors of [30] found that “porcine blood samples showed the most abundant red blood cell, hemoglobin concentration, packed cell volume and plasma protein content as well as its freeze-dried blood possessed the highest contents of protein, fat, Cu and Cr with the highest percentage of heme iron (*p* < 0.05)” [30]. In conclusion, the authors of [30] highlighted that information concerning specific blood parameters and their correlations for selected animal breeds (including pigs) could be used for producing of “food supplementation or product development based on their potential applications” [30].

From the other hand, the determination of relationships (including the numerical correlations) between biochemical parameters and productive indicators in animals is of great fundamental and practical importance. For example, Novopashina S.I. and co-authors [31] established the relationship between animal conformation and productivity (up to 0.68), hematological parameters and productivity (up to 0.51) for dairy goats. According to the authors [31], this is of great importance in selection and breeding work. Moreover, these correlations are recommended for application in creative selection of the highly productive herds of dairy goats [25,31,32,33]. For example, the authors [33] found that “hematological and biochemical parameters were widely correlated with animal health biomarkers in various pathologies” [33]. Amino acids, which are the main nutrients for pigs, are not only digested and used by the host for the synthesis of protein and other important substances, but also have additional functions in relation to growth, health and disease [34,35]. For example, proline as one of the most abundant amino acids in colostrum and milk of pigs, plays an extremely important role “in the perception of cellular energy, cell differentiation, etc. and may contribute to a decrease in the redox status of the cell [36]. The authors of [37] summarized numerous studies of the biochemical composition of various animal organs and tissues, as well as the general relationships between the major biochemical parameters (including amino acids) of animal biological fluids “to determine the biomarkers of various disorders and abnormalities in the physiological state” [37]. In a more recent publication [38], the authors studied the numerical correlations between the quality indicators of beef meat and the protein profile of the muscle tissue as “the effect of animal’s pre-slaughter stress” [38]. The authors of [39] studied the correlations between the content of microflora and the biochemical status of the contents of the large intestine. The obtained results proved that “dietary symbiotic supplementation can improve piglet’s survival and lipid metabolism by altering gut microbiota diversity and composition in pregnant and lactating sow’s” [39]. Thus, a detailed study of the correlations between the amino acid composition and biochemical parameters of Duroc pig blood will be actual and useful both from fundamental and applied points.

The aims of the work were the following: to measure the amino acids and biochemical parameters of the pig blood serum; and to establish the major correlations of all the obtained parameters in dependence on the fattening duration.

## 2. Results and Discussion

### 2.1. The Total Amount of Amino Acids in Pig Blood Serum vs. the Fattening Duration

The measurements of the total amount of amino acids (AAs) in pig blood serum (depending on the fattening duration) were obtained and presented in the first part of our original research. It is important to highlight that the average values for 15 from 20 major AAs led to a visual change in the obtained data set (summarized in the Table 1).

It is believed that for a comprehensive analysis it is better to use median values (as one of the most reliable and stable indicators), rather than mean (arithmetic averages), which can significantly distort reality. For example, if one indicator is very different from the others in the selection, then it seriously distorts the final result. In the last case the differences between median and mean values can be more than few orders. In all cases (shown in the Table 1) the differences between median and mean values are mainly in the range of 5–7% (10%—maximum in the Table 1). Thus, no indicator regarding the content of specific amino acids (Table 1) differs decidedly from the rest (i.e., there is not a single value that stands out greatly from the total mass of values in the selection).

It is important to highlight that pronounced differences were found between the studied groups. In most cases, descriptive statistics in the “R” program showed a high reliability of differences in the presented parameters (Table 1) between these groups of animals: AB—reliability between the first and second group; AC—reliability between the first and third group; AD—reliability between the first and fourth group; BC—reliability between the second and third group; BD—reliability between the second and fourth groups; CD—reliability between the third and fourth groups.

**Figure 1:**
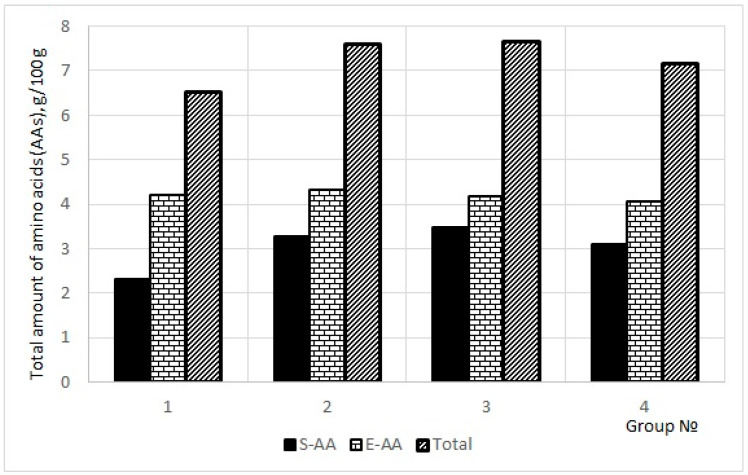
The amount of synthesizable amino acids (S-AA), the amount of essential amino acids (E-AA) and the total amount of amino acids (Total) in pig blood serum in each of the four groups (n = 80) depending on the fattening duration (65–90 days): *X*-axis—animal groups, *Y*-axis—the content of S-AA (left black columns); E-AA (middle “stone” hatching columns); total AA (right “oblique” hatching columns).

The maximum average (mean) values were found for such AAs as GLU 1.05–1.14 (72–82 days), LEU 0.78–0.80 (72–82 days), LYS 0.72 (72 days), and ASP 0.71 (82 days) in pig blood serum (Table 1). The minimum average (mean) values were found for such AAs as ILE 0.23, HIS 0.23 and GLY 0.23 (65 days in all cases) according to the Table 1. Some of these data are a lit bit over the range of the previously published data for other animals [18,19,20]. In contrast, a majority of the average (mean) AA values (found for all AAs, especially in the pig groups 2–4) were almost in the range of the published data for another pig breeds [16,40]. If calculating the changes in the relative values of the particular AAs (Figure 1) were especially pronounced in the case of long fattening duration (groups 3 and 4, i.e., 82–90 days) as compared to the short one (groups 1 and 2, i.e., 65–72 days presented in the Table 1).

The amount of synthesizable amino acids (S-AA) in pig blood serum in each of the four groups (Figure 1) was the following: (1) 2.32 (35.6%); (2) 3.27 (43.1%); (3) 3.48 (45.4%); (4) 3.09 (43.2%). The amount of essential amino acids (E-AA) in pig blood serum in each of the four groups was the following: (1) 4.20 (64.4%); (2) 4.31 (56.9%); (3) 4.18 (54.6%); (4) 4.06 (56.8%). The total amount of amino acids (Total) in pig blood serum in each of the four groups was the following (g/100 g): (1) 6.52; (2) 7.58; (3) 7.66; (4) 7.15. The ratios of essential to synthesizable amino acids (E-AA/S-AA) in each of the animal group were the following: (1) 1.81; (2) 1.32; (3) 1.20; (4) 1.32. All these data (Figure 1) proved an increase in amino acid metabolism in the groups two to four (fattening duration 72–90 days).

To our opinion, the ratios of essential to synthesizable amino acids (E-AA/S-AA) in each of the animal group is more informative for the possible state standards of the reference values for husbandry practice, as compared to the separate E-AA or S-AA values, or the percentage to the total amount of amino acids in pig blood serum.

### 2.2. The Major Biochemical Parameters of the Blood Serum vs. the Fattening Duration

The measurements of the major biochemical parameters of the blood serum (depending on the fattening duration) were obtained and presented in the second part of our research. The average values for the major biochemical parameters were in the range of the published data for another pig breeds [16,25], but had the pronounced changes in the obtained data set summarized below. 

Analyzing the state of nitrogen metabolism in the body of pigs, we noted significant differences in the concentration of total protein (74.1 and 71.5 g/L) between animals of the groups 1 (65 days of fattening) and 4 (90 days of fattening) (*p* < 0.05). This may be due to the fact that with the age of animals, the intensity of protein metabolism and the use of protein for muscle tissue synthesis are slowly decreasing (but in the range of the physiological norms). Changes in the concentration of protein fractions (albumins, globulins) are subject to the same pattern. For example, in the group 2 (72 days of fattening) the content of albumins was about 42.0 g/L, which is significantly higher than those (37.8 g/L) of the group 4 (*p* < 0.01). The contents of urea, the final metabolite of nitrogen metabolism, in the blood of pigs of the groups 4 (8.65 mM) and 3 (8.46 mM) were significantly higher as compared to the animal groups 1 (7.62 mM, *p* < 0.05) and 2 (7.28 mM, *p* < 0.01), which indicated a lower level of use of nitrogenous substances in the blood of pigs with age (by the fattening duration).

The change in the concentration of lipid metabolism metabolites (increase in the level of cholesterol and triglycerides with age) in the studied blood is interconnected with an increase in the deposition of adipose tissue of animals with an increase in the number of days of fattening. For example, the TG contents in the blood of pigs of the groups 4 (0.91 mM) and 3 (0.85 mM) were significantly higher as compared to the animal groups 1 and 2 (0.28 mM, *p* < 0.01 in the both cases).

With an increase in the duration of fattening of animals, the “load” on the liver increases, and therefore the activity of ALT in the blood of animals significantly increases. For example, the ALT contents in the blood of pigs of the groups 3 (31.9 U/L) and 4 (30.1 U/L) were significantly higher as compared to the animal group 1 (22.7 U/L, *p* < 0.001). The levels of TAWSA in the blood of pigs of groups 3 (15.42 U/L) and 4 (16.03 U/L) were significantly higher as compared to the animal group 1 (9.66 U/L, *p* < 0.001) that could be associated with a change in the levels of numerous water-soluble metabolites with antioxidant activities in the pig blood (as the parts of TAWSA value).

### 2.3. The Results of the Correlation Coefficients between AAs and All Parameters of the Pig Blood Serum in All Groups

The positive (negative) correlation coefficients (such as: ±0.75–±1.0 = very strong; ±0.50–±0.74 = strong; ±0.25–±0.49 = moderate; ±0.01–±0.24 = weak) were calculated between all obtained amino acids and the major biochemical parameters of the blood serum of Duroc pigs for the groups 1–4 and presented in the Table 2, Table 3, Table 4, Table 5, Table 6, Table 7, Table 8 and Table 9.

The relationships between the content of AAs and the number of proteins (blood albumins and globulins) have been especially important, since AAs are the building blocks of the protein molecules and the key elements that maintained the structure of proteins “due to hydrogen bonds and hydrophobic interactions” [3]. There is a clear stable positive relationship between the content of AAs and total protein (r = 0.64) in the blood serum. The correlation coefficient between albumins and ALA was positive moderate (0.46), which indicated a direct relationship between these parameters. The important joint role of amino acid ASP and phosphorus in the energy metabolism of all animals were established earlier, therefore the positive moderate correlation (r = 0.43) between them was expected. Positive correlations of AST (aspartate aminotransferase) with amino acids, in particular—with such amino acids as ASP (r = 0.54) and HIS (r = 0.51), can be associated with the process of AA oxidation to urea and carbon dioxide during metabolism. AA oxidation began with the removal of the amino group (via transaminase) and its incorporation into the urea cycle. Positive correlations between the content of some AAs and glucose: GLY (r = 0.42), ALA (r = 0.46), PHE (r = 0.53) were found. These correlations associated with the process of glycogenesis, during which glycogenic AAs “can be converted into glucose” [3].

High correlation coefficients for many AAs with urea: ASP (r = 0.72), GLU (r = 0.73), GLY (r = 0.75), LYS (r = 0.74), ARG (r = 0.77)—can be related to the oxidation of AAs discussed above. Ammonia, formed during the catabolism of AAs in the intestine, either entered the portal vein or used locally for the synthesis of urea. The presence of a functional urea cycle in enterocytes served as “the first line of defense against ammonia toxicity in mammals” [3]. The presence of positive correlations between the content of calcium and some AAs, in particular, SER (r = 0.52), may be due to the fact that serine is a part of some enzymes involved in signal transmission of the nervous system. The presence of positive correlations between the content of ALT and such AAs as THR (r = 0.62), ALA (r = 0.61), TYR (r = 0.57) was due to transamination reactions catalyzed by this enzyme. The stable negative correlations between magnesium and AAs (from −0.67 to −0.91) can be explained by the fact that magnesium actively chelates with AAs. The strong and very strong correlation coefficients between the content of Ca and such AAs as ASP (r = 0.70), GLY (r = 0.73), PRO (r = 0.90)—were due to the fact that all these substances played a key role in the homeostasis of the animal body and also participated in a variety of metabolic processes.

At the same time, there are pronounced positive correlations between the content of AAs and calcium in the Duroc serum (coefficients: r = 0.63 for serine, r = 0.41 for valine, r = 0.43 for proline) and only weak correlations with phosphorus, magnesium, etc. Calcium, phosphorus and magnesium are the common mineral macronutrients for animals (as well as for humans) and involved in some biochemical processes in the cells [3]. It is especially calcium ions which involved in the key physiological processes, such as blood coagulation processes, serve as one of the universal secondary mediators inside cells and regulate a variety of intracellular processes (muscle contraction, exocytosis, secretion of hormones and neurotransmitters) [3]. That is why to study the calcium level in the blood is especially important. The strong and medium correlations of the calcium level in the blood can be explained by particular interactions (associations) of ionized Ca^2+^ with the blood proteins (mainly, with albumins).

Analyzing the correlations mentioned above, we can conclude that the animals of the studied groups were almost adapted to the keeping and fattening conditions, their metabolism and metabolic processes did not exceed the established norms. This is evidenced by stable moderate correlations between the content of AAs and total protein, albumins, globulins, and calcium in the pig blood plasma. The relationship between amino acid and biochemical parameters is the best traced in the last two groups of animals (82 and 90 days of fattening).

In order to evaluate the optimal fattening duration and metabolic changes the results of the correlation analysis between the major AAs and biochemical parameters of the pig blood serum were discussed the biochemical parameters in each of four animal groups separately.

### 2.4. The Correlation Coefficients between AAs and TASWA

It was found that in the majority of cases the value of the correlation coefficient between TASWA and AAs characterized by moderate values. For example, some correlations were found between TASWA and such AAs as ASP—0.46; THR 0.50; GLU 0.49; ALA 0.69; VAL—0.47; ILE 0.47; LEU 0.49; TYR 0.51; PHE 0.49; LYS 0.48; ARG—0.46; PRO—0.47. Estimation of the relationship between the amino acid composition of blood and its antioxidant activity can be of particular importance for the food industry, since these indicators directly linked with the quality and “shelf-life” of meat products.

### 2.5. The Correlation Coefficients between AAs and Protein Fractions or N-Metabolite’s Parameters

When comparing the major AA and biochemical parameters of the pig blood serum (as indicators of protein and nitrogen metabolism), it was concluded that there were a number of correlations of varying degrees between the studied parameters. It was clear that very strong, strong or moderate correlations were the most important and called “informative” as compared to low correlations (no matter, positive or negative by sign).

The total 51 “informative” correlations (from 60 max. possible) between the AA content and the 4 protein parameters were found in the pig blood for the first group of animals (65 days fattening, Table 2), whereas only very low correlations between the AA content and the nitrogen metabolites (such as urea and creatinine) were found (Table 2). In particular, the six moderate correlations (from 0.25 to 0.36) were observed between the major AAs and the total protein content in the pig blood serum for the first group of animals (Table 2). The eight strong and seven moderate correlations were observed between the major AAs and the albumins’ content in the pig blood serum (Table 2). The six strong and nine moderate correlations were observed between the major AAs and the globulins’ content in the pig blood serum (Table 2). The 1 very strong, 11 strong, and 3 moderate correlations were observed between the major AA content and the A/G ratios (i.e., albumins’ to globulins’) in the pig blood serum (Table 2). Thus, all 15 AAs studied had “informative” correlations with the 3 protein parameters, whereas such six AAs as THR, GLY, ALA, VAL, ILE, PRO, had the “informative” correlations with all 4 protein parameters.

There were only very low correlations (not “informative”) found between the AA content and the protein parameters in the pig blood for the second group of animals (72 days fattening, Table 4). The exception was only PRO content that correlated at the moderate level with the globulins (0.26), creatinine (0.31) and ALP (0.30) in the pig blood for this animal group (Table 4). Thus, only three moderate correlations (from 270 max. possible) between the AA content and all biochemical parameters were found in the pig blood for the second group of animals (72 days fattening, Table 4).

The total 36 “informative” correlations (from 60 max. possible) between the AA content and the 4 protein parameters were found in the pig blood for the third group of animals (82 days fattening, Table 6)., whereas only 12 correlations (1 strong and 11 moderate) between the AA content and the nitrogen metabolites (such as urea and creatinine) were found (Table 6). In particular, seven strong and five moderate correlations were observed between the major AAs and the total protein content in the pig blood serum for the third group of animals (Table 6). The only five moderate correlations were observed between the major AAs and the albumins’ content in the pig blood serum (Table 6). The five strong and seven moderate correlations were observed between the major AAs and the globulins’ content in the pig blood serum (Table 6). The two strong and five moderate correlations were observed between the major AA content and the A/G ratios in the pig blood serum (Table 6).

The total 47 “informative” correlations (from 60 max. possible) between the AA content and the protein parameters were found in the pig blood for the 4th group of animals (90 days fattening, Table 8). In particular, 14 (9 very strong and 5 strong) correlations between the AA content and the total protein content in the pig blood serum for this group of animals (Table 8). The two strong and nine moderate correlations were observed between the major AAs and the albumins’ content in the pig blood serum (Table 8). The 13 strong and 1 moderate correlations were observed between the major AAs and the globulins’ content in the pig blood serum (Table 8). The only eight moderate correlations were observed between the major AA content and the A/G ratios in the pig blood serum (Table 8).

The 2 very strong, 11 strong, and 1 moderate correlations were observed between the major AAs and the urea level were found, whereas only very low correlations were found with creatinine level in the pig blood for the 4th group of animals (90 days fattening, Table 8).

Urea synthesized in the liver and in the rumen wall from ammonia nitrogen, amino acids, amides, and considered as the “end product” of nitrogen metabolism for all mammals, including pigs [15,16,17]. Urea usually accounted for at least half of the animal residual nitrogen in the blood and 80–83% of those in urine [15,16,17]. A decrease in the concentration of urea in the blood of animals indicated an increase in protein metabolism in their body [16,17]. In addition, urea is also a representative of the class of low molecular weight antioxidants [18,19,20].

Thus, the total amount of the “informative” correlations (of AA vs. total protein and its fractions) for each group, was the following: 51, 47, 36 and 1 for the groups 1, 4, 3, 2 (Table 2, Table 8, Table 6 and Table 4, respectively). The total amount of the “informative” correlations (of AA vs. some nitrogen metabolites) for each group was the following: 14, 12, 1 and 0 for the groups 4, 3, 2, 1 (Table 8, Table 6, Table 4 and Table 2, respectively).

### 2.6. The Correlation Coefficients between AAs and Glucose Level

The total 15 strong correlations (from 15 max. possible) between the AA content and the glucose level (as carbohydrates parameters) were found in the pig blood for the first group of animals (65 days fattening, Table 2).

In contrast, there were no “informative” correlations between the AA content and the glucose level correlations found in the pig blood for the second group of animals (72 days fattening, Table 4).

There were one strong and five moderate correlations found between the AA content and the glucose level in the pig blood for the third group of animals (82 days fattening, Table 6). The only four moderate correlations between the AA content and the glucose level in the pig blood were found for the fourth group of animals (90 days fattening, Table 8).

Thus, the majority of low level correlations with glucose levels can be reasonable, since the AAs have different metabolic pathways with numerous metabolites that are not always converting to glucose.

Thus, the total amount of the “informative” correlations (of AA vs. glucose) for each group, was the following: 15, 6, 4 and 0 for the groups 1, 3, 4, 2 (Table 2, Table 6, Table 8 and Table 4, respectively).

### 2.7. The Correlation Coefficients between AAs and Lipid Parameters

The total 14 moderate correlations (from 30 max. possible) between the AA content and the triglycerides (TG) and cholesterol (Chol) levels (as lipid parameters) were found in the pig blood for the first group of animals (65 days fattening, Table 2). In contrast, there were absolutely no “informative” correlations found between the AA content and triglycerides and cholesterol levels in the pig blood for the second group of animals (72 days fattening, Table 4). The total 6 (7) correlations between the AA content and the triglycerides (cholesterol) levels were found in the pig blood for the third group of animals (82 days fattening, Table 6). The 2 strong and 11 moderate correlations between the AA content and the triglycerides, but only 2 moderate correlations—with cholesterol levels were found in the pig blood for the fourth group of animals (90 days fattening, Table 8).

Thus, the large amount of low level and medium correlations with lipid parameters can be reasonable, since the AA have different metabolic pathways with numerous metabolites that are rarely converting to lipids. The total amount of the “informative” correlations (of AA vs. some lipids such as triglycerides and cholesterol) for each group, was the following: 28, 15, 13 and 0 for the groups 1, 3, 4, 2 (Table 2, Table 6, Table 8 and Table 4, respectively).

### 2.8. The Correlation Coefficients between AAs and Some Enzyme’s Parameters

The total 28 “informative” correlations (from 60 max. possible) between the AA content and the following enzyme parameters: 14 moderate with AST and 13 moderate with AST/ALT ratio, but only 1 with ALP, were found in the pig blood for the first group of animals (65 days fattening, Table 3). In contrast, only very weak correlations (below 0.25) were observed between the major AA and the ALT content in the pig blood serum for the first group of animals (Table 3). There was only very 1 informative” correlation found between the AA content and the four enzyme parameters in the pig blood for the second group of animals (72 days fattening, Table 5). There were 33 informative” correlations found between the AA content and the following enzyme parameters: 3 strong and 8 moderate—with AST, 4 moderate—with ALT, 10 moderate—with AST/ALT ratio and 8 moderate—with ALP in the pig blood for the third group of animals (82 days fattening, Table 7). The total 30 “informative” correlations (from 60 max. possible) were found between the AA content and the following enzyme parameters: 8 strong and 6 moderate—with ALT, 1 moderate—with AST, 1 strong and 12 moderate—with AST/ALT ratio and 1 moderate—with ALP in the pig blood for the 4th group of animals (90 days fattening, Table 9).

Thus, the total amount of the “informative” correlations (of AA vs. some enzymes) for each group was the following: 33, 30, 28 and 1 for the groups 3, 4, 1, 2 (Table 7, Table 9, Table 3 and Table 5, respectively). These correlations supported some previous data [25,26,27,28,29,30,31,32,33,34,35,36,37,38,39,40] concerning the regulatory effects of amino acid levels on the activity of some enzymes.

## 3. Materials and Methods

### 3.1. Samples of the Pig Blood Serum

The studies were carried out on boars of the Duroc breed (n = 80 total animals) grown at the feeding stations of the selection-hybrid center (SHC, Russia). All animals were divided onto the 4 animal groups: (1) 65 fattening days (n = 12), (2) 72 fattening days (n = 36), (3) 82 fattening days (n = 19), (4) 90 fattening days (n = 13), respectively. All studied animals were clinically healthy. In accordance with the accepted technology of keeping at SHC, the feeding of animals was carried out after the completion of the rearing period, while the live weight of piglets varied within 25–36 kg. The animal live weight at the beginning of fattening was the following: group 1, 35.0 ± 0.34 kg; group 2, 30.3 ± 0.32 kg; group 3, 33.5 ± 0.35 kg; group 4, 35.4 ± 0.29 kg. Live weight when removed from the feed station: group 1—98.21 ± 1.03 kg; group 2—110.05 ± 1.03; group 3—111.75 ± 1.02; group 4—110.52 ± 1.20. The stations were completed as the batch of animals changed at the end of the test period. Automatic feed distribution was designed for animals weighing from 25 to 140 kg (during the control rearing period) and the diet was the same for the entire studied boar population and was divided according to the growth period:

- period 1 (from 1 to 21 days): dry matter—80%, metabolizable energy—about 3500 kcal, crude protein—16.70%, crude fat—4.38%, crude fiber—4.39%, lysine—1.11%, methionine + cysteine—0.67%, calcium—0.55% and phosphorus—0.52%;
- period 2 (from 22 to 35 days): dry matter—80%, metabolizable energy—about 3300 kcal, crude protein—14.59%, crude fat—3.57%, crude fiber—4.12%, lysine—0.95%, methionine + cysteine—0.58%, calcium—0.55% and phosphorus—0.48%;
- period 3 (from 36 days to the end of fattening): dry matter—80%, metabolizable energy—about 3250 kcal, crude protein—13.10%, crude fat—2.17%, crude fiber—4.49%, lysine—0.83%, methionine + cysteine—0.51%, calcium—0.51% and phosphorus—0.49%.

The blood samples were taken from the ear vein at the end of the fattening. The serum was separated by centrifugation for 15 min at 3000 rpm/min (laboratory centrifuge SM-12, Russia).

The experimental protocols (concerning these animals) were approved by the Ethics Committee of the Federal Research Center for Animal Husbandry named after Academy Member L.K. Ernst (protocol code: 2021–2303; date of approval: 23 March 2021). All experiments and conditions (animal care, feeding, biological material sampling, etc.) were fulfilled in accordance with the applicable regulations (internationally recognized guidelines and local acts).

### 3.2. Measurements of the Biochemical Indicators of Pig Blood Serum Samples

The biochemical indicators of animal blood serum sample were determined using a “ChemWell” automatic biochemical analyzer (Awareness Technology, USA) with reagents of “Analyticon Biotechnologies AG” (Germany) and “Spinreact” (Spain). The following biochemical indicators [40]: the concentration of total protein (TP)—by biuret method; albumin (A)—by colorimetric method; urea—by enzymatic colorimetric (Berthelot method); creatinine—by the kinetic Yaffe method; glucose—by enzymatic glucose oxidase method; cholesterol (Chol) and triglycerides (TG) by enzyme-colorimetric method; bilirubin (quantification by Walters and Gerarde method); calcium (Ca) by O-cresolphthalein complexon method; phosphorus (P), magnesium (Mg) and iron (Fe) by colorimetric method; alanine aminotransferase (ALT) activity by UV-kinetic method; aspartate aminotransferase (AST) activity by UV-kinetic method; alkaline phosphatase (ALP) activity by kinetic method were determined. The following ratios and indicators: A/G, Ca/P, ALT/AST and the concentration of globulins (G) were determined by calculation [40]. The results of these measuring were statistically processed using the “R” program

### 3.3. Measurements of the Amino Acid Content of Pig Blood Serum Samples

Exactly 200 μL of each sample of the pig blood serum was selected and added to 3 mL of 6 N hydrochloric acid (HCl) for complete hydrolysis. The hydrolysis was carried out in fluoroplastic beakers with a screw cap (CEM, Corporation, Matthews, NC, USA), in a thermostat at 110 °C for 24 h. Norleucine was used as an internal standard for AA analysis. During such hydrolysis (6 H HCI, 24 h, 110 °C) a tryptophan was completely destroyed, whereas asparagines and glutamine were converted to the corresponding acids. In turn, cysteine and methionine at such conditions were oxidized to cysteic acid and methionine sulfone, respectively, and excluded from the further consideration.

After hydrolysis, 160 μL of the resulting suspension was taken and evaporated at 110 °C to remove hydrochloric acid. Then, 1 mL of sample dilution buffer was added. The resulting suspension was centrifuged at 13,000 rpm for 5 min. The final amino acid analysis was carried out using an LC-20 Prominence high performance liquid chromatography system (Shimadzu, Tokyo, Japan) equipped with a reaction module for post-column derivatization with ninhydrin ARM-1000 (Sevko & Co., Moscow, Russia) and a column with ion exchange resin 4.6 × 150 mm (Sevko & Co., Moscow, Russia).

The correlations between the AA content and major biochemical parameters of the pig blood serum (the concentration of total protein and its major fractions, carbohydrates, lipids, nitrogen metabolites, as well as the activity of some enzymes in the pig’s blood for each of the animal groups) were fulfilled using statistical treatment by “R” program.

### 3.4. Amperometric Method for Measurements of the Total Amount of Water-Soluble Antioxidants in the Pig Blood Serum Samples

The amperometric method [41,42,43,44] was used to study the total amount of the water-soluble antioxidants (TAWSA). The measurements were carried out on a “Tsvet-Yauza 01-AA” device [41,42]. The TAWSA values were determined by measuring the strength of the electric current arising during the oxidation of molecules on the surface of the working electrode at a potential ~500 mV. TAWSA was measured in equivalent to gallic acid as reference [41]. For this, “working solutions” were prepared from a gallic acid solution (100 mg/dm^3^) for calibration with a mass concentration of 0.2; 0.5; 1.0 and 4.0 mg/dm^3^. Phosphoric acid solution 2.2 mmol/dm^3^ was used as an “eluent” [42]. The results of measuring the total antioxidant activity of the samples were statistically processed using the “R” program.

The obtained datasets of the amino acids, antioxidant and biochemical parameters of the Duroc breed boars is deposited at the web-site of the L.K. Ernst Federal Research Center for Animal Husbandry (https://www.vij.ru/institut/struktura-organizatsii/nauchnye-podrazdeleniya/gruppa-analiticheskoj-biokhimii-2 (accessed on 1 January 2022) and the relevant accession number will be provided. 

## 4. Conclusions

An estimation of the major AA and biochemical parameters of the blood serum of Duroc pig of the Russian population is an important point in assessing the range of the reference values for husbandry practice. In our opinion, the ratios of essential to synthesizable amino acids (E-AA/S-AA) in each of the animal group is more informative for the possible state standards of the reference values for husbandry practice, as compared to the separate E-AA or S-AA values, or the percentage to the total amount of amino acids in pig blood serum. On the other hand, the authors proposed for consideration (for the possible inclusion in the state standards) the numerous sets of correlations between the major AA and biochemical parameters depending on the fattening duration that were obtained for the first time.

Based on the total amount of such correlations for each group, the authors proposed the following data of the total amount of correlation coefficients (with reasonable *p*-values) in each of the group separately: group 1, 1* (*p* < 0.05); group 2, 0; group 3, 28* (*p* < 0.05) and 9** (*p* < 0.01); group 4, 28* (*p* < 0.05) and 25** (*p* < 0.01). These correlation values of AAs vs. major biochemical parameters of the pig blood serum found for the first time.

Thus, about 82–90 days (groups 3 and 4) can be the optimal time for pig fattening based on the correlation analysis for the numerous data of major AA and biochemical parameters of pig blood serum. These results can be useful for animal health monitoring and husbandry.

## Figures and Tables

**Table 1:** The total amount of amino acids (AAs) in pig blood serum in each of the four groups (n = 80) depending on the fattening duration (65–90 days).

AAs	Statistics	Group 1 (65 Days)	Group2 (72 Days)	Group3 (82 Days)	Group4 (90 Days)
		n = 12	n = 36	n = 19	n = 13
**ASP**	**Mean**	0.57 **^,AB^	**0.66**	0.71 ***^,AC^	0.64 **^,CD^
	SD	0.15	0.11	0.10	0.07
	SEM	0.04	0.02	0.02	0.02
	Median	0.62	0.70	0.69	0.67
	Kurtosis	−0.05	0.28	0.29	1.31
**THR**	**Mean**	0.35 **^,AB^	**0.41**	**0.40**	**0.38**
	SD	0.10	0.07	0.05	0.04
	SEM	0.03	0.01	0.01	0.01
	Median	0.38	0.43	0.40	0.39
	Kurtosis	−0.13	0.13	−0.38	1.11
**SER**	**Mean**	0.33 **^,AB^	**0.41**	0.43 **^,AC^	**0.42**
	SD	0.13	0.07	0.11	0.14
	SEM	0.04	0.01	0.02	0.04
	Median	0.37	0.43	0.45	0.40
	Kurtosis	2.05	−0.18	0.02	10.55
**GLU**	**Mean**	0.90 **^,AB^	**1.05**	1.14 ***^,AC^	0.99 ***^,CD^
	SD	0.23	0.17	0.12	0.11
	SEM	0.07	0.03	0.03	0.03
	Median	0.99	1.12	1.11	1.04
	Kurtosis	0.12	0.65	−1.16	1.58
**GLY**	**Mean**	**0.23**	0.25 **^,BC^	0.29 **^,AC^	**0.27**
	SD	0.06	0.04	0.05	0.03
	SEM	0.02	0.01	0.01	0.01
	Median	0.25	0.27	0.27	0.28
	Kurtosis	−0.07	0.01	−0.62	1.84
**ALA**	**Mean**	0.47 **^,AB^	0.56 ***^,BC^	**0.44**	0.45 ***^,BD^
	SD	0.12	0.10	0.07	0.03
	SEM	0.04	0.02	0.02	0.01
	Median	0.51	0.59	0.44	0.46
	Kurtosis	0.65	0.63	−0.03	1.84
**VAL**	**Mean**	**0.42**	**0.47**	**0.45**	**0.45**
	SD	0.11	0.08	0.06	0.05
	SEM	0.04	0.01	0.01	0.01
	Median	0.46	0.50	0.45	0.47
	Kurtosis	−0.18	0.06	0.96	0.63
**ILE**	**Mean**	0.23 **^,AB^	**0.27**	**0.28**	**0.25**
	SD	0.06	0.04	0.13	0.03
	SEM	0.02	0.01	0.03	0.01
	Median	0.26	0.28	0.26	0.27
	Kurtosis	0.12	0.35	16.09	1.58
**LEU**	**Mean**	0.68 **^,AB^	**0.78**	**0.77**	**0.74**
	SD	0.17	0.12	0.17	0.08
	SEM	0.05	0.02	0.04	0.02
	Median	0.75	0.82	0.82	0.77
	Kurtosis	0.04	0.45	7.82	1.09
**TYR**	**Mean**	0.37 **^,AB^	**0.44**	0.43 **^,AC^	**0.41**
	SD	0.10	0.07	0.05	0.05
	SEM	0.03	0.01	0.01	0.01
	Median	0.41	0.47	0.44	0.44
	Kurtosis	0.30	0.42	−1.00	1.76
**PHE**	**Mean**	0.42 **^,AB^	**0.48**	**0.49**	**0.46**
	SD	0.11	0.08	0.11	0.05
	SEM	0.03	0.01	0.03	0.01
	Median	0.46	0.51	0.49	0.47
	Kurtosis	0.27	0.52	11.30	1.34
**HIS**	**Mean**	0.23 **^,AB^	0.26 ***^,BC^	0.31 ***^,AC^	0.26 **^,CD^
	SD	0.05	0.04	0.06	0.03
	SEM	0.02	0.01	0.01	0.01
	Median	0.24	0.27	0.30	0.27
	Kurtosis	0.05	0.51	1.63	0.22
**LYS**	**Mean**	0.63 **^,AB^	**0.72**	**0.65**	**0.67**
	SD	0.16	0.12	0.10	0.07
	SEM	0.05	0.02	0.02	0.02
	Median	0.70	0.75	0.68	0.70
	Kurtosis	0.08	0.81	−0.07	1.86
**ARG**	**Mean**	**0.41**	0.47 ***^,BC^	**0.39**	0.44 **^,CD^
	SD	0.11	0.09	0.07	0.05
	SEM	0.04	0.01	0.02	0.02
	Median	0.45	0.49	0.39	0.46
	Kurtosis	−0.13	0.53	1.52	2.41
**PRO**	**Mean**	0.29 **^,AB^	0.34 ***^,BC^	0.48 *** **^,^** **^AC^**	0.32 ***^,CD^
	SD	0.07	0.06	0.10	0.04
	SEM	0.02	0.01	0.02	0.01
	Median	0.31	0.35	0.48	0.32
	Kurtosis	−0.17	−0.05	0.70	−0.17

**Mean values** are the major numbers and in bold; SD, standard deviation; SEM, standard error of the mean; C_V_, coefficient of variation. *—*p* < 0.05; **—*p* < 0.01; ***—*p* < 0.001. ^AB^—reliability between the first and second group; ^AC^—reliability between the first and third group ^AD^—reliability between the first and fourth group; ^BC^—reliability between the second and third group; ^BD^—reliability between the second and fourth groups; ^CD^—reliability between the third and fourth groups.

**Table 2:** The correlation coefficients between all obtained amino acids and the major biochemical parameters of the blood serum of Duroc pigs of the group 1 (65 days). TP, total protein; A, albumins; G, globulins; A/G, albumins to globulins ratio, urea, creatinine, glucose; TG, triglycerides; Chol, cholesterol (n = 12).

AAs^1^	TP, g/L	A, g/L	G, g/L	A/G, r.u.	Urea, mM	Creatinine, µM	Glucose, mM	TG, mM	Chol, mM
**ASP**	0.24	−0.51	0.49	−0.55	−0.03	0.05	−0.72 **	−0.33	−0.27
**THR**	0.35	−0.53	0.59 *	−0.61 *	−0.11	0.05	−0.69 *	−0.43	−0.25
**SER**	0.28	−0.67 *	0.62 *	−0.76 **	−0.04	−0.03	−0.69 *	−0.45	−0.41
**GLU**	0.20	−0.48	0.44	−0.52	0.02	0.06	−0.68 *	−0.33	−0.28
**GLY**	0.36	−0.53	0.60 *	−0.61 *	−0.15	0.08	−0.71 *	−0.41	−0.25
**ALA**	0.03	−0.46	0.30	−0.43	0.20	0.17	−0.60 *	−0.15	−0.28
**VAL**	0.30	−0.55	0.56	−0.61 *	−0.07	0.06	−0.70 *	−0.38	−0.24
**ILE**	0.25	−0.50	0.50	−0.56	0.01	0.05	−0.67 *	−0.36	−0.27
**LEU**	0.20	−0.47	0.44	−0.51	0.04	0.04	−0.69 *	−0.32	−0.27
**TYR**	0.23	−0.53	0.49	−0.57	0.02	0.08	−0.65 *	−0.37	−0.29
**PHE**	0.18	−0.49	0.44	−0.53	0.06	0.05	−0.66 *	−0.34	−0.28
**HIS**	0.21	−0.44	0.43	−0.50	0.02	0.10	−0.65 *	−0.32	−0.28
**LYS**	0.17	−0.44	0.40	−0.47	0.07	0.04	−0.65 *	−0.31	−0.25
**ARG**	0.18	−0.42	0.39	−0.46	0.00	0.04	−0.71 *	−0.29	−0.28
**PRO**	0.30	−0.56	0.57	−0.62 *	−0.10	0.08	−0.72 **	−0.38	−0.28

Notes AAs^1^: Asp, aspartic acid; Thr, threonine; Ser, serine; Glu, glutamic acid; Gly, glycine; Ala, alanine; Val, valine; Ile, isoleucine; Leu, leucine; Tyr, tyrosine; Phe, phenylalanine; His, histidine; Lys, lysine; Arg, arginine; Pro, proline; *—*p* < 0.05; **—*p* < 0.01; ***—*p* < 0.001. **These values** are the major numbers.

**Table 3:** The correlation coefficients between all obtained amino acids and the major biochemical parameters of the blood serum of Duroc pigs of the group one (65 days): ALT, alanine aminotransferase; AST, aspartate aminotransferase; ALT/AST, alanine to aspartate aminotransferases’ ratio; Ca, calcium; P, phosphorus; Ca/P, calcium to phosphorus ratio; Mg, magnesium; TAWSA, total amount of the water-soluble antioxidants (n = 12).

AAs	ALT, U/L	AST, U/L	AST/ALT, r.u.	ALP, U/L	Ca, mM/L	P, mM/L	Ca/P, r.u.	Mg, mM/L	TAWSA, r.u.
**ASP**	0.01	−0.34	−0.30	−0.20	0.39	0.15	0.08	0.17	−0.33
**THR**	0.05	−0.31	−0.29	−0.22	0.39	0.09	0.12	0.13	−0.32
**SER**	−0.12	−0.20	−0.13	−0.31	0.63 *	−0.13	0.39	0.22	−0.34
**GLU**	0.00	−0.34	−0.29	−0.17	0.38	0.17	0.06	0.16	−0.34
**GLY**	0.05	−0.32	−0.30	−0.20	0.40	0.08	0.13	0.14	−0.33
**ALA**	−0.13	−0.37	−0.26	−0.08	0.35	0.13	0.07	0.20	−0.31
**VAL**	0.03	−0.32	−0.29	−0.20	0.41	0.11	0.12	0.17	−0.32
**ILE**	0.02	−0.33	−0.29	−0.21	0.40	0.13	0.09	0.19	−0.34
**LEU**	0.00	−0.32	−0.27	−0.18	0.36	0.18	0.04	0.16	−0.33
**TYR**	−0.02	−0.36	−0.31	−0.19	0.38	0.10	0.11	0.15	−0.31
**PHE**	−0.03	−0.33	−0.27	−0.18	0.38	0.14	0.08	0.14	−0.31
**HIS**	0.04	−0.32	−0.30	−0.13	0.38	0.15	0.08	0.14	−0.32
**LYS**	0.00	−0.30	−0.25	−0.15	0.34	0.19	0.02	0.13	−0.31
**ARG**	−0.01	−0.26	−0.21	−0.14	0.33	0.17	0.03	0.07	−0.29
**PRO**	0.02	−0.36	−0.33	−0.20	0.43	0.10	0.13	0.20	−0.36

*—*p* < 0.05; **—*p* < 0.01; ***—*p* < 0.001. **These values** are the major numbers.

**Table 4:** The correlation coefficients between all obtained amino acids and the major biochemical parameters of the blood serum of Duroc pigs of the group 2 (72 days): TP, total protein; A, albumins; G, globulins; A/G, albumins to globulins ratio, urea, creatinine, glucose; TG, triglycerides; Chol, cholesterol (n = 36).

AAs^1^	TP, g/L	A, g/L	G, g/L	A/G, r.u.	Urea, mM	Creatinine, µM	Glucose, mM	TG, mM	Chol, mM
**ASP**	0.11	0.13	0.05	−0.01	0.08	0.12	0.00	−0.05	−0.06
**THR**	0.07	0.11	0.02	0.02	0.01	0.10	−0.02	−0.03	−0.09
**SER**	0.13	0.15	0.07	0.00	0.06	0.15	0.00	−0.04	−0.05
**GLU**	0.09	0.13	0.03	0.00	0.06	0.11	0.00	−0.06	−0.06
**GLY**	0.10	0.15	0.03	0.02	0.05	0.12	−0.03	−0.05	−0.05
**ALA**	0.16	0.13	0.11	−0.09	0.14	0.13	0.05	−0.04	−0.08
**VAL**	0.04	0.09	0.00	0.02	0.00	0.06	−0.02	−0.05	−0.06
**ILE**	0.02	0.10	−0.05	0.05	0.01	0.06	−0.03	−0.02	−0.06
**LEU**	0.05	0.10	0.00	0.01	0.07	0.10	0.00	−0.01	−0.02
**TYR**	0.06	0.12	−0.01	0.02	0.04	0.10	−0.01	−0.02	−0.05
**PHE**	0.03	0.09	−0.02	0.02	0.05	0.08	0.00	−0.02	−0.02
**HIS**	−0.06	0.07	−0.11	0.06	0.02	0.01	−0.02	−0.03	−0.05
**LYS**	−0.08	0.06	−0.13	0.09	−0.01	0.01	−0.05	−0.05	−0.04
**ARG**	−0.06	0.08	−0.13	0.10	0.00	0.00	−0.08	−0.05	−0.03
**PRO**	0.23	0.05	0.26	−0.22	0.18	0.31	0.15	0.24	0.11

**Table 5:** The correlation coefficients between all obtained amino acids and the major biochemical parameters of the blood serum of Duroc pigs of the group 2 (72 days): ALT, alanine aminotransferase; AST, aspartate aminotransferase; ALT/AST, alanine to aspartate aminotransferases’ ratio; Ca, calcium; P, phosphorus; Ca/P, calcium to phosphorus ratio; Mg, magnesium; TAWSA, total amount of the water-soluble antioxidants (n = 36).

AAs	ALT, U/L	AST, U/L	AST/ALT, r.u.	ALP, U/L	Ca, mM/L	P, mM/L	Ca/P, r.u.	Mg, mM/L	TAWSA, r.u.
**ASP**	0.01	−0.04	−0.04	−0.02	0.01	0.21	−0.17	−0.01	0.12
**THR**	−0.01	−0.06	−0.04	−0.02	−0.03	0.19	−0.17	−0.04	0.08
**SER**	−0.01	−0.06	−0.05	−0.01	−0.03	0.22	−0.20	0.00	0.11
**GLU**	0.03	−0.02	−0.02	−0.05	−0.01	0.19	−0.16	0.00	0.09
**GLY**	0.04	−0.06	−0.06	0.00	−0.01	0.24	−0.20	−0.01	0.10
**ALA**	0.00	−0.08	−0.08	−0.06	0.01	0.20	−0.17	0.04	0.07
**VAL**	0.00	−0.07	−0.05	0.00	0.00	0.17	−0.14	−0.06	0.06
**ILE**	0.03	0.00	0.01	0.03	0.01	0.15	−0.11	−0.06	0.03
**LEU**	0.02	−0.01	0.00	0.06	0.02	0.17	−0.13	−0.04	0.04
**TYR**	0.02	−0.02	−0.02	0.03	−0.01	0.17	−0.14	−0.04	0.02
**PHE**	0.02	−0.02	−0.01	0.05	0.01	0.16	−0.13	−0.06	0.03
**HIS**	−0.02	0.05	0.09	0.08	0.08	0.11	−0.03	−0.09	−0.04
**LYS**	0.00	0.04	0.07	0.05	0.04	0.09	−0.05	−0.11	−0.06
**ARG**	−0.01	0.06	0.09	0.06	0.04	0.10	−0.06	−0.09	−0.05
**PRO**	−0.03	−0.14	−0.13	0.30	−0.08	0.18	−0.21	−0.03	0.17

**Table 6:** The correlation coefficients between all obtained amino acids and the major biochemical parameters of (82 days): TP, total protein; A, albumins; G, globulins; A/G, albumins to globulins ratio, urea, creatinine, glucose; TG, triglycerides; Chol, cholesterol (n = 19).

AAs	TP, g/L	A, g/L	G, g/L	A/G, r.u.	Urea, mM	Creatinine, µM	Glucose, mM	TG, mM	Chol, mM
**ASP**	0.50 *	0.34	0.40	−0.15	−0.02	−0.42	0.22	0.38	0.52 *
**THR**	0.53 *	0.24	0.48 *	−0.27	0.24	−0.01	−0.09	0.08	0.18
**SER**	0.06	−0.09	0.12	−0.13	−0.30	−0.25	0.41	0.03	0.33
**GLU**	0.65 **	0.15	0.68 **	−0.55 *	−0.03	−0.27	−0.09	0.55 *	0.35
**GLY**	0.51 *	−0.09	0.65 **	−0.61 **	−0.18	−0.24	0.32	0.44	0.39
**ALA**	0.52 *	0.40	0.38	−0.13	−0.10	−0.67 **	0.29	0.48 *	0.17
**VAL**	0.55 *	0.25	0.50 *	−0.28	0.01	−0.35	0.12	0.23	0.10
**ILE**	0.06	0.06	0.03	−0.03	0.17	0.08	−0.51 *	0.08	−0.11
**LEU**	0.43	0.24	0.38	−0.18	−0.01	−0.20	0.20	0.09	0.10
**TYR**	0.58 **	0.16	0.59 **	−0.40	0.34	−0.04	−0.23	0.10	0.04
**PHE**	0.32	0.20	0.26	−0.11	−0.11	−0.30	0.40	0.11	0.28
**HIS**	0.49 *	0.10	0.53 *	−0.40	−0.24	−0.42	0.35	0.46 *	0.46 *
**LYS**	0.45	0.27	0.38	−0.19	0.06	−0.36	0.21	0.36	0.35
**ARG**	0.32	0.24	0.24	−0.06	0.27	−0.16	0.17	0.10	0.34
**PRO**	0.15	−0.28	0.33	−0.46 *	−0.38	−0.08	0.12	0.13	0.23

*—*p* < 0.05; **—*p* < 0.01; ***—*p* < 0.001. **These values** are the major numbers.

**Table 7:** The correlation coefficients between all obtained amino acids and the major biochemical parameters of the blood serum of Duroc pigs of group three (82 days): ALT, alanine aminotransferase; AST, aspartate aminotransferase; ALT/AST, alanine to aspartate aminotransferases’ ratio; Ca, calcium; P, phosphorus; Ca/P, calcium to phosphorus ratio; Mg, magnesium; TAWSA, total amount of the water-soluble antioxidants (n = 19).

AAs	ALT, U/L	AST, U/L	AST/ALT, r.u.	ALP, U/L	Ca, mM/L	P, mM/L	Ca/P, r.u.	Mg, mM/L	TAWSA, r.u.
**ASP**	0.27	0.64 **	0.40	−0.25	−0.14	0.40	−0.42	0.07	−0.08
**THR**	0.14	0.45	0.31	−0.46 *	−0.17	0.08	−0.09	−0.21	−0.24
**SER**	0.07	0.47 *	0.43	−0.17	−0.05	0.15	−0.24	−0.09	0.03
**GLU**	0.14	0.34	0.27	−0.23	−0.29	0.16	−0.15	0.36	0.08
**GLY**	0.29	0.55 *	0.25	−0.30	−0.13	0.28	−0.30	0.35	−0.08
**ALA**	0.31	0.44	0.15	−0.20	0.07	0.51 *	−0.43	0.19	−0.21
**VAL**	0.41	0.48 *	0.01	−0.34	−0.07	0.42	−0.38	−0.04	−0.52 *
**ILE**	−0.02	−0.16	−0.16	0.14	−0.04	−0.07	0.03	0.11	0.41
**LEU**	0.05	0.40	0.35	−0.47 *	0.02	0.21	−0.13	−0.14	−0.38
**TYR**	0.12	0.22 **	0.08	−0.40	−0.07	0.07	−0.04	0.19	−0.17
**PHE**	0.17	0.42	0.26	−0.27	0.01	0.26	−0.22	0.01	−0.39
**HIS**	0.19	0.61	0.41	−0.30	−0.07	0.48 *	−0.51 *	0.28	−0.04
**LYS**	0.02	0.44	0.47 *	−0.22	−0.16	0.32	−0.37	0.11	−0.16
**ARG**	0.15	0.23	0.09	−0.06	−0.11	0.19	−0.20	0.17	−0.52 *
**PRO**	−0.06	0.22	0.35	−0.21	−0.07	−0.09	0.05	0.22	0.46 *

*—*p* < 0.05; **—*p* < 0.01; ***—*p* < 0.001. **These values** are the major numbers.

**Table 8:** The correlation coefficients between all obtained amino acids and the major biochemical parameters of the blood serum of Duroc pigs of the group 4 (90 days): TP, total protein; A, albumins; G, globulins; A/G, albumins to globulins ratio, urea, creatinine, glucose; TG, triglycerides; Chol, cholesterol (n = 13).

AAs	TP, g/L	A, g/L	G, g/L	A/G, r.u.	Urea, mM	Creatinine, µM	Glucose, mM	TG, mM	Chol, mM
**ASP**	0.79 **	0.37	0.61 *	−0.32	0.72 **	0.01	0.16	−0.41	0.14
**THR**	0.74 **	0.16	0.69 **	−0.48	0.68 **	0.04	0.12	−0.34	0.08
**SER**	0.15	0.20	0.04	0.06	0.28	0.11	0.32	0.03	0.46
**GLU**	0.77 **	0.47	0.52	−0.21	0.73 **	−0.02	0.17	−0.41	0.15
**GLY**	0.77 **	0.43	0.55	−0.23	0.75 **	−0.14	0.11	−0.56 *	0.00
**ALA**	0.64 *	0.13	0.60 *	−0.46	0.21	0.24	−0.09	−0.01	0.29
**VAL**	0.73 **	0.29	0.59 *	−0.34	0.68 *	−0.01	0.25	−0.39	0.05
**ILE**	0.72 **	0.42	0.51	−0.22	0.74 **	−0.07	0.21	−0.42	0.07
**LEU**	0.79 **	0.48	0.54	−0.22	0.71 **	−0.03	0.18	−0.43	0.13
**TYR**	0.78 **	0.40	0.58 *	−0.29	0.73 **	0.01	0.08	−0.40	0.13
**PHE**	0.79 **	0.44	0.57 *	−0.26	0.72 **	−0.02	0.17	−0.43	0.12
**HIS**	0.76 **	0.54	0.48	−0.14	0.68 *	−0.10	0.32	−0.55	0.04
**LYS**	0.79 **	0.53	0.51	−0.18	0.74 **	0.03	0.07	−0.38	0.20
**ARG**	0.78 **	0.46	0.55	−0.25	0.77 **	0.06	0.03	−0.39	0.18
**PRO**	0.67 *	0.23	0.57 *	−0.34	0.60 *	−0.05	0.27	−0.34	0.05

*—*p* < 0.05; **—*p* < 0.01; ***—*p* < 0.001. **These values** are the major numbers.

**Table 9:** The correlation coefficients between all obtained amino acids and the major biochemical parameters of the blood serum of Duroc pigs of the group 4 (90 days): ALT, alanine aminotransferase; AST, aspartate aminotransferase; ALT/AST, alanine to aspartate aminotransferases’ ratio; Ca, calcium; P, phosphorus; Ca/P, calcium to phosphorus ratio; Mg, magnesium; TAWSA, total amount of the water-soluble antioxidants (n = 13).

AAs	ALT, U/L	AST, U/L	AST/ALT, r.u.	ALP, U/L	Ca, mM/L	P, mM/L	Ca/P, r.u.	Mg, mM/L	TAWSA, r.u.
**ASP**	0.47	−0.06	−0.35	−0.11	0.41	0.03	0.26	−0.38	−0.66 *
**THR**	0.62 *	0.09	−0.24	−0.03	0.33	0.06	0.20	−0.37	−0.61 *
**SER**	−0.23	−0.48	−0.58 *	−0.24	0.52	0.38	−0.04	−0.04	−0.30
**GLU**	0.48	−0.09	−0.40	−0.15	0.44	0.07	0.23	−0.40	−0.63 *
**GLY**	0.48	−0.02	−0.29	−0.34	0.26	−0.20	0.39	−0.38	−0.75 **
**ALA**	0.61 *	0.25	−0.03	0.03	−0.01	0.21	−0.18	−0.13	−0.05
**VAL**	0.59 *	0.03	−0.31	−0.11	0.42	0.10	0.20	−0.32	−0.65 *
**ILE**	0.53	−0.04	−0.37	−0.12	0.47	0.10	0.22	−0.43	−0.62 *
**LEU**	0.48	−0.07	−0.36	−0.17	0.41	0.04	0.24	−0.36	−0.65 *
**TYR**	0.57 *	0.00	−0.32	−0.20	0.29	0.00	0.21	−0.34	−0.63 *
**PHE**	0.54	−0.03	−0.34	−0.18	0.38	0.02	0.25	−0.37	−0.65 *
**HIS**	0.49	−0.12	−0.42	−0.29	0.47	−0.02	0.33	−0.39	−0.69 **
**LYS**	0.49	−0.07	−0.36	−0.16	0.34	0.03	0.21	−0.36	−0.59 *
**ARG**	0.54	−0.04	−0.34	−0.16	0.28	−0.02	0.21	−0.35	−0.60 *
**PRO**	0.50	0.00	−0.31	0.00	0.49	0.16	0.19	−0.38	−0.57 *

*—*p* < 0.05; **—*p* < 0.01; ***—*p* < 0.001. **These values** are the major numbers.

## Data Availability

The obtained datasets of the amino acids, antioxidant and biochemical parameters of the Duroc breed boars is deposited at the web-site of the L.K. Ernst Federal Research Center for Animal Husbandry (https://www.vij.ru/institut/struktura-organizatsii/nauchnye-podrazdeleniya/gruppa-analiticheskoj-biokhimii-2 (accessed on 1 January 2022) and the relevant accession number will be provided.

## References

[1] Chalvon-Demersay T., Luise D., Le Floc’h N., Tesseraud S., Lambert W., Bosi P., Trevisi P., Beaumont M., Corrent E. (2021). Functional amino acids in pigs and chickens: Implication for gut health. Front. Vet. Sci..

[2] Akram M., Asif M., Uzair M., Naveed M., Madni A., Asadullah M., Ali S., Zahoor H., Asmat K. (2011). Amino acids: A review article. J. Med. Plants Res..

[3] Zaitsev S.Y. (2017). Biological Chemistry: From Biologically Active Substances to Organs and Tissues of Animals.

[4] Karau A., Grayson I. (2014). Amino acids in human and animal nutrition. Adv. Biochem. Eng. Biotechnol..

[5] Wu G., Bazer F.W., Burghardt R.C., Johnson G.A., Kim S.W., Knabe D.A., Li X.L., Satterfield M.C., Smith S.B. (2010). Functional amino acids in swine nutrition and production. Dyn. Anim. Nutr..

[6] Iltyakov A.V., Mikolaichik I.N., Morozova L.A., Stupina E.S. (2015). The method of increasing the biological usefulness of muscle and adipose tissue of pigs. Agrar. Bull. Urals.

[7] Bulatov A.P., Mikolaichik I.N. (2006). Influence of natural sorbents on product quality and natural resistance of young pigs. Rep. Russ. Acad. Agric. Sci..

[8] Neupokoeva A.S., Iltyakov A.V. (2019). Amino acid composition of meat of pigs of different genotypes. Actual Probl. Sci. Support Dev. Mod. Anim. Husb..

[9] Morozova L.A., Mikolaychik I.N., Il’tyakov A.V., Duskaev G.K. (2019). Amino acid composition of muscle tissue of purebred and hybrid pigs in the continental climate of Russia. Agrar. Bull. Ural..

[10] Zhang S., Zeng X., Ren M., Mao X., Qiao S. (2017). Novel metabolic and physiological functions of branched chain amino acids: A review. J. Anim. Sci. Biotechnol..

[11] Wang J., Chen L., Li P., Li X., Zhou H., Wang F., Li D., Yin Y., Wu G. (2008). Gene expression is altered in piglet small intestine by weaning and dietary glutamine supplementation. J. Nutr..

[12] Zayko O.A., Korotkevich O.S., Petukhov V.L. (2013). The content of macro- and microelements in the liver of pigs of early maturing meat breed (SM-1) and their relationship with the level of free amino acids in blood serum. Rep. Russ. Acad. Agric. Sci..

[13] Zhang F., Zheng W., Xue Y., Yao W. (2019). Suhuai suckling piglet hindgut microbiome-metabolome responses to different dietary copper levels. Appl. Microbiol. Biotechnol..

[14] Zayko O.A., Mager S.N. The content of copper in the liver of pigs of early maturing meat breed (SM-1) and its correlation with the amino acid profile of the blood. Proceedings of the International Scientific Online Conference “AgroNauka-2020”.

[15] Kulintsev V.V., Osmanova S.O., Omarov M.O. (2011). Demand for lysine in young pigs. Agrar. Sci..

[16] Zaitsev S.Y. (2016). Tensiometric and Biochemical Analysis of Animal Blood: Fundamental and Applied Aspects.

[17] Knorre D.G., Myzina S.D. (2000). Biological Chemistry.

[18] Petukhova M.A. (2016). Amino acid composition and biological value of meat of pigs of various genotypes. Rep. Natl. Acad. Sci. Belarus.

[19] Fedorenkova L.A., Sheiko R.I., Yanovich E.A., Petukhova M.A., Khramchenko N.M., Burnos A.C., Batkovskaya T.V. (2015). Muscle amino acid content tissues of young pigs of different breeds. Zootech. Sci. Belarus.

[20] Habibi M., Shili C., Sutton J., Goodarzi P., Maylem E.R., Spicer L., Pezeshki A. (2021). Branched-chain amino acids partially recover the reduced growth of pigs fed with protein-restricted diets through both central and peripheral factors. Anim. Nutr..

[21] Almeida F.N., Htoo J.K., Thomson J., Stein H.H. (2013). Comparative amino acid digestibility in US blood products fed to weanling pigs. Anim. Feed. Sci. Technol..

[22] Brotzge S.D., Chiba L.I., Adhikari C.K., Stein H.H., Rodning S.P., Welles E.G. (2014). Complete replacement of soybean meal in pig diets with hydrolyzed feather meal with blood by amino acid supplementation based on standardized lleal amino acid digestibility. Livest. Sci..

[23] National Research Council (2012). Nutrient Requirements of Swine.

[24] (2017). Meat and Meat Products. Determination of Amino Acids Composition of Animal Protein.

[25] Wang J., Xiao Y., Li J., Qi M., Tan B. (2021). Serum biochemical parameters and amino acids metabolism are altered in piglets by early-weaning and proline and putrescine supplementations. Anim. Nutr..

[26] Yu J., Liu G., Zhang J., Zhang C., Fan N., Xu Y., Guo J., Yuan J. (2021). Correlation among serum biochemical indices and slaughter traits, texture characteristics and water-holding capacity of Tan sheep. Ital. J. Anim. Sci..

[27] Klimienė I., Špakauskas V., Matusevičius A. (2005). Correlation of Different Biochemical Parameters in Blood Sera of Healthy and Sick Cows. Vet. Res. Commun..

[28] Akashi N., Azuma K., Tsuka T., Kawamoto H., Amaha T., Yamashita M., Osaki T., Ito N., Okamoto Y., Murahata Y. (2019). Correlations between states of amino acids and hematology or plasma biochemistry in calves within 24 hours after birth. Anim. Husb. Dairy Vet. Sci..

[29] Abeni F., Petrera F., Prà A.D., Rapetti L., Crovetto G.M., Galassi G. (2018). Blood parameters in fattening pigs from two genetic types fed diet with three different protein concentrations. Transl. Anim. Sci..

[30] Sorapukdee S., Narunatsopanon S. (2017). Comparative Study on Compositions and Functional Properties of Porcine, Chicken and Duck Blood. Korean J. Food Sci. Anim. Resour..

[31] Novopashina S.I., Kondrashina I.V. (2015). Correlation of Traits in Dairy Goats of the Saanen Breed. Collect. Sci. Work. All-Russ. Sci. Res. Inst. Sheep Goat Breed..

[32] Dijkstra A.M., van Vliet N., van Vliet D., Romani C., Huijbregts S.C., van der Goot E., Hovens I.B., van der Zee E.A., Kema I.P., Heiner-Fokkema M.R. (2021). Correlations of blood and brain biochemistry in phenylketonuria: Results from the Pah-enu2 PKU mouse. Mol. Genet. Metab..

[33] Blázquez R., Álvarez V., Antequera-Barroso J.A., Báez-Díaz C., Blanco V., Maestre J., Sánchez-Margallo F.M. (2018). Altered hematological, biochemical and immunological parameters as predictive biomarkers of severity in experimental myocardial infarction. Vet. Immunol. Immunopathol..

[34] Liu Y., Wang X., Hou Y., Yin Y., Qiu Y., Wu G., Hu C.-A.A. (2017). Roles of amino acids in preventing and treating intestinal diseases: Recent studies with pig models. Amino Acids.

[35] Yang Z., Liao S.F. (2019). Physiological effects of dietary amino acids on gut health and functions of swine. Front. Vet. Sci..

[36] Kang P., Zhang L., Hou Y., Ding B., Yi D., Wang L., Zhu H., Liu Y., Yin Y., Wu G. (2014). Effects of L-proline on the growth performance, and blood parameters in weaned lipopolysaccharide (LPS)-challenged pigs Asian. Australas. J. Anim. Sci..

[37] Kaneko J.J., Harvey J.W., Bruss M.L. (2008). Clinical Biochemistry of Domestic Animals.

[38] Díaz F., Díaz-Luis A., Sierra V., Diñeiro Y., González P., García-Torres S., Tejerina D., Romero-Fernández M.P., de Vaca M.C., Coto-Montes A. (2020). What functional proteomic and biochemical analysis tell us about animal stress in beef. J. Proteom..

[39] Ma C., Zhang W., Gao Q., Zhu Q., Song M., Ding H., Yina Y., Kong X. (2020). Dietary synbiotic alters plasma biochemical parameters and fecal microbiota and metabolites in sows. J. Funct. Foods.

[40] Zaitsev S.Y., Bogolyubov N.V., Zhang X., Brenig B. (2020). Biochemical parameters, dynamic tensiometry and circulating nucleic acids for cattle blood analysis. PeerJ.

[41] Yashin Y.I., Ryzhnev V.Y., Yashin A.Y., Chernousov N.I. (2009). Natural Antioxidants. Content in Food Products and Their Impact on Human Health and Aging.

[42] Yashin A.Y. (2008). Injector-Flow System with Amperometric Detector for selective determination of antioxidants in meal additives and drinks. Russ. Chem. J..

[43] Savina A.A., Voronina O.A., Bogolyubov N.V., Zaitsev S.Y. (2020). Amperometric detection of antioxidant activity of model and biological fluids. Mosc. Univ. Chem. Bull..

[44] Zaitsev S.Y., Savina A.A., Volnin A.A., Voronina O.A., Bogolyubova N.V. (2020). Comparative study of the water-soluble antioxidants in fodder additives and sheep blood serum by amperometric and biochemical methods. Animals.

